# Electron Acceptor Availability Shapes Anaerobically Methane Oxidizing Archaea (ANME) Communities in South Georgia Sediments

**DOI:** 10.3389/fmicb.2021.617280

**Published:** 2021-04-14

**Authors:** Annika Schnakenberg, David A. Aromokeye, Ajinkya Kulkarni, Lisa Maier, Lea C. Wunder, Tim Richter-Heitmann, Thomas Pape, Petra Pop Ristova, Solveig I. Bühring, Ingrid Dohrmann, Gerhard Bohrmann, Sabine Kasten, Michael W. Friedrich

**Affiliations:** ^1^Microbial Ecophysiology Group, Faculty of Biology/Chemistry, University of Bremen, Bremen, Germany; ^2^International Max Planck Research School of Marine Microbiology, Max Planck Institute for Marine Microbiology, Bremen, Germany; ^3^MARUM – Center for Marine Environmental Sciences, University of Bremen, Bremen, Germany; ^4^Faculty of Geosciences, University of Bremen, Bremen, Germany; ^5^Hydrothermal Geomicrobiology Group, MARUM – Center for Marine Environmental Sciences, University of Bremen, Bremen, Germany; ^6^Alfred Wegener Institute Helmholtz Centre for Polar and Marine Research, Bremerhaven, Germany

**Keywords:** anaerobic oxidation of methane, marine sediment, anaerobic methane-oxidizing archaea, methane hydrates, microbial community analysis, ANME-1-related, ANME-2a

## Abstract

Anaerobic methane oxidizing archaea (ANME) mediate anaerobic oxidation of methane (AOM) in marine sediments and are therefore important for controlling atmospheric methane concentrations in the water column and ultimately the atmosphere. Numerous previous studies have revealed that AOM is coupled to the reduction of different electron acceptors such as sulfate, nitrate/nitrite or Fe(III)/Mn(IV). However, the influence of electron acceptor availability on the *in situ* ANME community composition in sediments remains largely unknown. Here, we investigated the electron acceptor availability and compared the microbial *in situ* communities of three methane-rich locations offshore the sub-Antarctic island South Georgia, by Illumina sequencing and qPCR of *mcrA* genes. The methanic zone (MZ) sediments of Royal Trough and Church Trough comprised high sulfide concentrations of up to 4 and 19 mM, respectively. In contrast, those of the Cumberland Bay fjord accounted for relatively high concentrations of dissolved iron (up to 186 μM). Whereas the ANME community in the sulfidic sites Church Trough and Royal Trough mainly comprised members of the ANME-1 clade, the order-level clade “ANME-1-related” (Lever and Teske, 2015) was most abundant in the iron-rich site in Cumberland Bay fjord, indicating that the availability of electron acceptors has a strong selective effect on the ANME community. This study shows that potential electron acceptors for methane oxidation may serve as environmental filters to select for the ANME community composition and adds to a better understanding of the global importance of AOM.

## Introduction

Anaerobic oxidation of methane (AOM) is of major importance to earth’s climate as it regulates the release of the methane from marine sediments into the hydrosphere and eventually into the atmosphere, where it can serve as a potent greenhouse gas (e.g., Jørgensen and Boetius, 2007; Reeburgh, 2007). In marine sediments, more than 90% of biogenic methane, which accounts for 7–25% of the global methane production, is oxidized to CO_2_ by AOM (Knittel and Boetius, 2009). The process is generally linked to sulfate reduction (S-AOM) producing a distinct sulfate-methane transition (SMT) in sediment layers where downward diffusing sulfate from seawater is reduced by upward diffusing methane (e.g., Iversen and Jørgensen, 1985; Niewöhner et al., 1998; Knittel and Boetius, 2009).

S-AOM is mediated by consortia of sulfate-reducing bacteria (SRB) and anaerobic methanotrophic archaea (ANME) (Boetius et al., 2000). ANME is a phylogenetically non-monophyletic group within the class of Methanomicrobia, consisting mainly of the order ANME-1 and the families ANME-2a/2b/2c, as well as the ANME-3 group within the order Methanosarcinales (Knittel and Boetius, 2009). Although ANME remain uncultivated, highly enriched cultures comprising candidate species of the family Methanoperedenaceae [formerly ANME-2d group or AOM-associated archaea (AAA)] have been obtained from freshwater sediments in bioreactors fed with nitrate and methane (e.g., by Haroon et al., 2013). These have been shown to link AOM not to sulfate but to alternative electron acceptors such as nitrate or metal oxides, i.e., Fe(III) (*Candidatus* “*Methanoperedens nitroreducens*,” Ettwig et al., 2016; *Ca.* “*Methanoperedens ferrireducens*” Cai et al., 2018) and Mn(IV) (*Ca.* “*Methanoperedens manganireducens*,” *Ca.* “*Methanoperedens manganicus*,” Leu et al., 2020). Their genomes encompass pathways for methane oxidation as well as multiple multi-heme cytochromes (Ettwig et al., 2016), which are generally regarded as an indicator for metal-oxide reduction pathways in cultivated Fe(III) reducing bacteria, e.g., *Shewanella oneidensis* and *Geobacter metallireducens* (Weber et al., 2006; Shi et al., 2007). This suggests that members of the Methanoperedenaceae can mediate metal oxide dependent-AOM without bacterial partners such as sulfate-reducing bacteria (e.g., Desulfobulbaceae and Desulfobacteraceae) that are required for S-AOM (Knittel and Boetius, 2009). Similarly, genome sequences of the phylotypes ANME-1 and ANME-2a derived from marine environments have been shown to contain the genetic blueprint for iron oxide dependent AOM (Fe-AOM) or manganese oxide dependent AOM (Mn-AOM) which encompasses genes encoding multi-heme cytochromes (Wang et al., 2014; McGlynn et al., 2015).

Multiple studies suggest that several marine sediment environments could be active Fe-AOM and Mn-AOM sites, based on the presence of geochemical prerequisites such as large quantities of buried reactive Fe(III) and/or Mn(IV) oxides as well as abundant methane (Hensen et al., 2003; Riedinger et al., 2005, 2014, 2017; März et al., 2008; Sivan et al., 2011; Egger et al., 2015, 2016a,b, 2017; Oni et al., 2015; Rooze et al., 2016; Luo et al., 2020). Direct proof for Fe-AOM in marine sediment by measuring rates has recently been provided for methanic, iron oxide-rich sediments of the North Sea using ^14^C-based short term incubations with and without inhibition of sulfate reduction (Aromokeye et al., 2020). Different ANME subtypes have been shown to co-occur in marine sediments; however, they may appear in distinct ecological niches (Knittel et al., 2005; Timmers et al., 2015). For instance, the concentrations of certain pore-water constituents apparently may act as environmental controls on the ANME community facilitating the niche formation. In cold seep sediments from the Norwegian continental slope, ANME-2a and ANME-2b predominated the upper sediments, which were low in sulfide (along with low methane concentrations). In contrast, ANME-2c were found to predominate in deeper sediments close to gas hydrates, where methane and sulfide concentrations were higher (Roalkvam et al., 2011). Moreover, several ANME subtypes have been suggested to adapt to both S-AOM and Fe-AOM, apparently depending on the availability of the respective electron acceptor (Takeuchi et al., 2011; Wankel et al., 2012; Aromokeye et al., 2020). In addition, electron acceptor availability and temperature have been suggested as crucial factors for shaping the microbial community composition in terrestrial mud volcanoes in the Junggar Basin (Ren et al., 2018). Furthermore, temperature, oxygen, sulfate penetration and methane concentration have been suggested as environmental controls for AOM in shallow marine sediments in the Eckernförde Bay, Baltic Sea (Treude et al., 2005). Despite recent progress, however, it remains uncertain to what extent the electron acceptor availability impacts the ANME community composition and how environmental factors shape the community in deep marine sediments.

**FIGURE 1:**
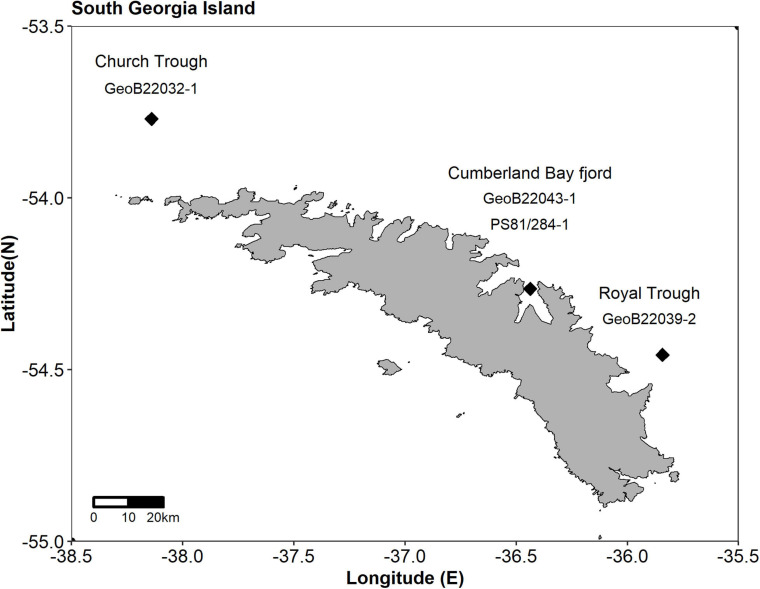
Map of South Georgia and sampling locations marked by diamonds. In west to east direction these are: Church Trough (GeoB22032-1), Cumberland Bay (GeoB22043-1, PS81/284-1) and Royal Trough (GeoB22039-2). In the Cumberland Bay fjord the two cores PS81/284-1 and GeoB22043-1 were taken in close proximity during two separate cruises as indicated by the black diamond.

The island of South Georgia is part of the North Scotia Ridge in the sub-Antarctic of the South Atlantic Ocean. The relatively broad shelf of the island is characterized by several large cross-shelf trough systems that likely formed during glacials and served as glacial outlets (Graham et al., 2017). The surrounding waters are characterized by large annual phytoplankton blooms (e.g., Borrione and Schlitzer, 2013). A recent hydroacoustic survey identified the northern shelf of South Georgia as an area of wide-spread active methane seepage related to trough systems (e.g., Römer et al., 2014). Here, we have investigated sediments from three geographically distant sampling sites, each located in a different trough/fjord system (Figure 1). We present a comprehensive geochemical and microbiological analysis revealing a potential Fe-AOM site in comparison with two sites, at which S-AOM potentially extends beyond the SMT into the MZ, of the methane-rich northern shelf of the sub-Antarctic South Georgia Island. We aim to identify specific ANME subgroups that are environmentally filtered by the availability of electron acceptors at the respective sites.

## Results

### Geochemical Characteristics of the Three Sampling Sites in the South Georgia Methane Seepage Area

**FIGURE 2:**
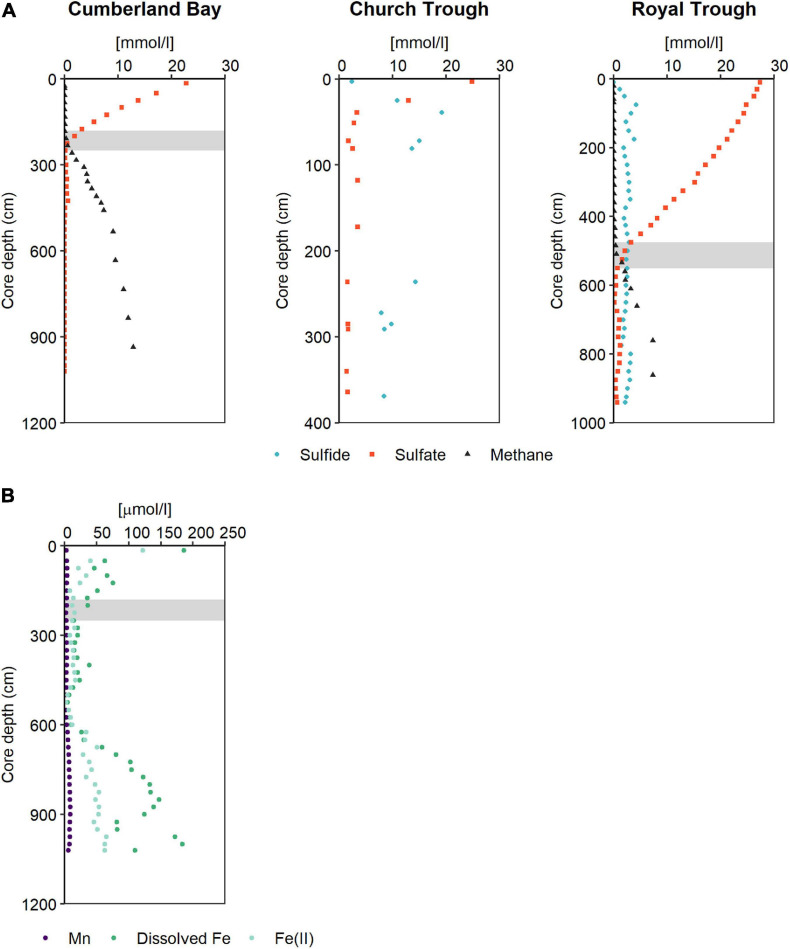
Pore-water profiles reflecting the geochemical prerequisites for and products of AOM in South Georgia sediments. **(A)** Pore-water profiles of sulfate, sulfide, and methane in the sediments of Cumberland Bay, Church Trough, and Royal Trough. **(B)** Pore-water profiles of ferrous iron, total dissolved iron and manganese in sediments of Cumberland Bay. Gray bars indicate SMT.

Three sites were sampled by gravity cores (GC) in Church Trough, Cumberland Bay fjord and Royal Trough during RV METEOR cruise M134 in 2017 (Bohrmann et al., 2017) along the northern shelf of South Georgia (Figure 1). Their main pore-water constituents (sulfate, sulfide, dissolved Fe and Mn, ferrous iron, and methane) were analyzed and compared (Figure 2). It was found that all study sites were abundant in methane (Figure 2A; Bohrmann et al., 2017) and dissolved inorganic carbon (DIC) (Supplementary Figure 1). The sediments of Royal Trough had abundant methane concentrations of up to 7 mM below 760 cm core depth. The sediment pore-water was sulfidic from 30 to 940 cm core depth (max. of 4 mM at 75 cm core depth) and sulfide was not restricted to the SMT (500 to 550 cm core depth) (Figure 2A). Both, sulfate and sulfide were present throughout the entire core except for the uppermost depth (10 cm, Figure 2A). Sulfate concentrations decreased downward from 27 mM at the top of the core to 1.5 mM at the SMT (475 to 550 cm) and varied between 0.3 and 1 mM in the MZ, whereas the sulfide concentrations varied between 1 and 4 mM throughout the entire core (Figure 2A). As both sulfide and sulfate were abundant throughout the core, the potential for S-AOM was not restricted to the SMT, but expanded into the MZ in Royal Trough sediments. Dissolved iron and manganese were only detected at 10 cm core depth where sulfide was absent (max. 34 μM and 1 μM, respectively) and not in the MZ (Supplementary Figure 2).

Both Church Trough and Royal Trough are located at the outer Northern shelf of South Georgia (Figure 1) and are characterized by highly sulfidic pore-water conditions. They are thus referred to as “sulfidic sites,” subsequently. Dissolved sulfate and sulfide were present over the entire core depth, however, sulfide concentrations were up to 4-fold higher in Church Trough (max. of 19 mM at 39 cm core depth, Figure 2A) compared to Royal Trough. The sulfate concentration decreased with depth from 27 to 2 mM (Figure 2A). The core from Church Trough (GeoB22032-1, Figure 1) comprised varying amounts of irregularly dispersed methane hydrates (Bohrmann et al., 2017). The gas hydrate saturation was high and reached a pore volume of 7.5% below 120 cm core depth, therefore measurement of dissolved methane was omitted (Bohrmann et al., 2017). Due to the consistently high sulfide and sulfate concentrations as well as methane saturation over the entire core depth, the depth of the SMT could not be determined (Figure 2A).

In contrast, the pore-water samples of the sediments of Cumberland Bay fjord revealed overall high dissolved iron and manganese concentrations with max. concentrations of 186 μM and 9 μM in the MZ, respectively (Figure 2B). Both dissolved iron and manganese reached high concentrations in the MZ, however, they were present also within the SMT around 250 cm core depth (Figure 2B). Dissolved iron concentrations (filtered via rhizon samplers of 0.15 μm average pore size) were between 2- and 70-fold higher than dissolved manganese concentrations (Figure 2B). Dissolved iron largely consisted of reduced Fe^2+^ (Figure 2B). Sulfate decreased downward from the top of the core into the SMT, and below detection limit in the MZ (<50 μM, Figure 2A). Methane was abundant below the SMT and reached a max. concentration of 13 mM at 935 cm depth (Figure 2A). Free sulfide was not detected in the sediments of the site in Cumberland Bay fjord due to the high dissolved iron concentrations present throughout the core and particularly in the MZ. Cumberland Bay fjord is hereinafter referred to as the “iron-rich site.”

### Microbial Community Composition and β-Diversity of Three Potential AOM Sites

In order to identify microorganisms potentially involved in S-AOM at the sulfidic sites Church Trough and Royal Trough or Fe/Mn-AOM at the iron-rich site Cumberland Bay fjord, the *in situ* microbial community compositions were studied at various sediment depths at all three sampling sites. In addition to the three aforementioned GC sampled during RV METEOR cruise M134 in 2017 (Bohrmann et al., 2017), a GC sampled during RV POLARSTERN cruise PS81 in 2013 in Cumberland Bay fjord (Bohrmann, 2013) was analyzed for the *in situ* microbial community composition. The bacterial communities were investigated mainly for the presence of known sulfate and iron reducing taxa among the Deltaproteobacteria. Desulfarculales, Desulfobacterales, and Desulfobulbaceae were present at all three sampling sites with max. relative abundances at the top sediments decreasing with depth (Supplementary Figures 3–5). Quantitative PCR of the *dsrA* gene confirmed the trend observed with Illumina sequencing (Supplementary Figure 6). The archaeal communities were most diverse with respect to the ANME subgroup distribution. The highest relative abundance of ANME 16S rRNA genes was found in Church Trough with a max. percentage of 51% of the total archaeal 16S rRNA gene sequences at 122 cm core depth (Supplementary Figure 4B). In Royal Trough, ANME made up a max. relative proportion of 30% of total archaeal 16S rRNA genes (Supplementary Figure 5B).

**FIGURE 3:**
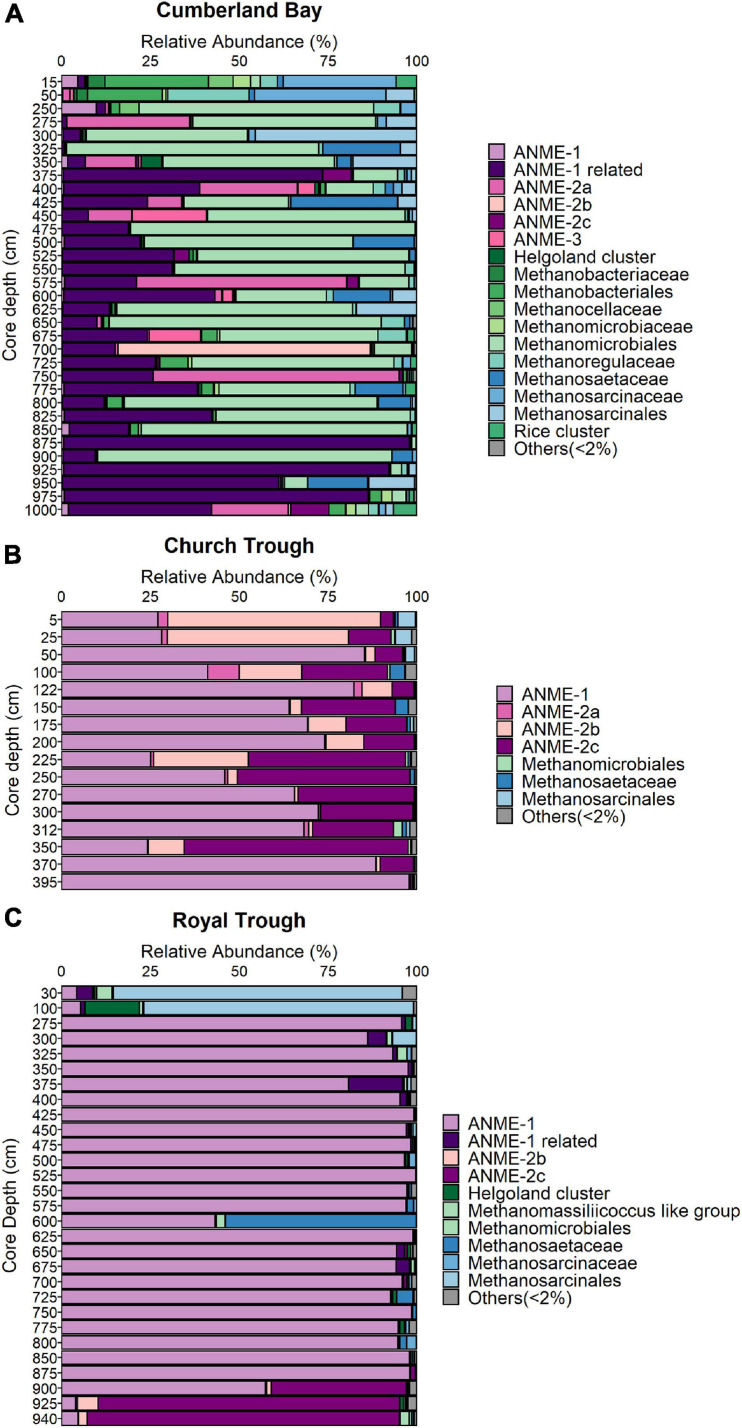
Distribution of ANME phylotypes in South Georgia sediments. Total sum scaling of relative abundances of the *mcrA* gene in **(A)** Cumberland Bay (GeoB22043-1), **(B)** Church Trough (GeoB22032-1), and **(C)** Royal Trough (GeoB22039-2).

**FIGURE 4:**
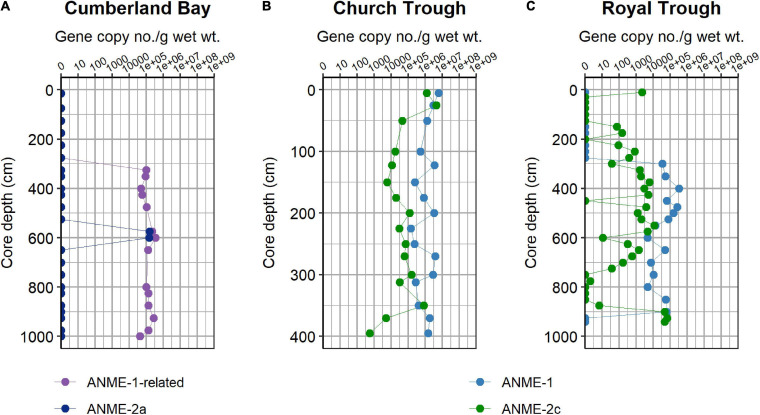
Abundance of ANME phylotypes in South Georgia sediments. Quantitative PCR analyses of *mcrA* gene copy numbers of **(A)** ANME-1-related and ANME-2a in Cumberland Bay, and *mcrA* copy numbers of ANME-1 and ANME-2c in **(B)** Church Trough and **(C)** Royal Trough.

The diversity of the ANME communities was examined in more detail by sequencing of the functional marker gene *mcrA* encoding the methyl coenzyme M reductase alpha subunit (Hales et al., 1996; Luton et al., 2002) which is a specific marker for methanogens and anaerobic methanotrophs (Luton et al., 2002; Friedrich, 2005). Illumina sequencing of the *mcrA* gene revealed that ANME-1 is the most abundant phylotype in both Church Trough (max. 98% at 395 cm core depth, Figure 3B) and Royal Trough (max. 99% at 425 cm core depth, Figure 3C) across most of sediment depths (Figures 3B,C). The second most abundant phylotypes in Church Trough were ANME-2b and ANME-2c (Figure 3B). ANME-2c also dominated the lowermost depths of Royal Trough sediments (Figure 3C). The abundance of ANME-1 was confirmed with qPCR in both Church Trough and Royal Trough (max. of 6 × 10^6^ and 4 × 10^5^ gene copies per gram wet sediment; Figures 4B,C, respectively).

**FIGURE 5:**
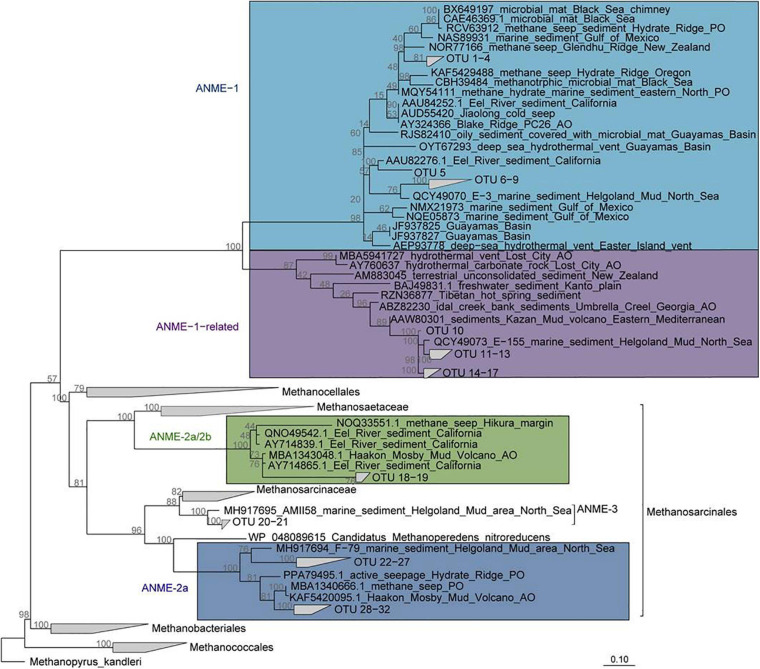
Phylogeny of ANME subgroups. Maximum likelihood tree (RAxML, 1000 Bootstraps) based on in-house *mcrA* gene database. OTU sequences of South Georgia were implemented with ARB parsimony. The ANME subgroups relevant to this study are highlighted: ANME-1-related (purple), ANME (light blue), ANME-2a (marine), and ANME-2c (green).

**FIGURE 6:**
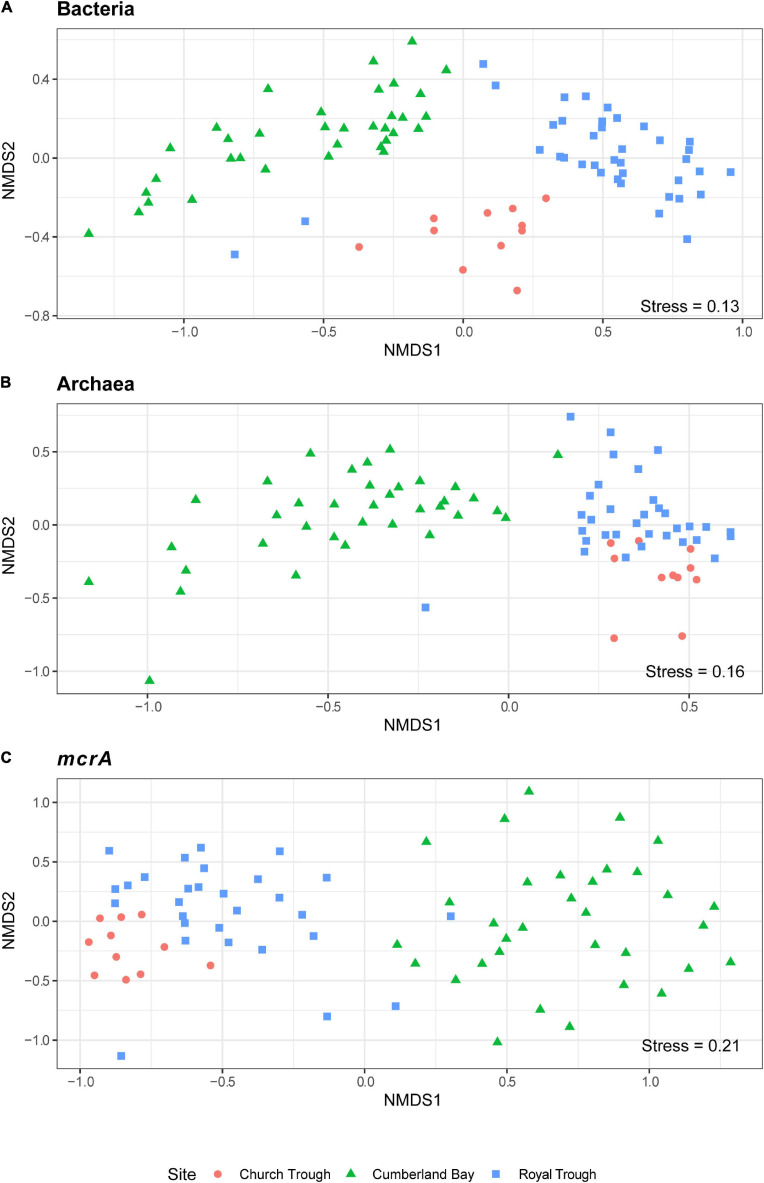
Site-specific β-diversity patterns of Cumberland Bay, Church Trough and Royal Trough. NMDS ordination of abundant OTUs (>1000 sequences) based on sequencing of the **(A)** bacterial 16S rRNA gene, **(B)** archaeal 16S rRNA gene, and **(C)** *mcrA* gene.

The archaeal community composition of Cumberland Bay fjord sediments differed greatly from the two sulfidic sites. Sequencing of the archaeal 16S rRNA gene revealed high relative abundances of ANME in the MZ (up to 17%), but very low in surface sediments (up to 0.1%) and SMT (up to 1%, Supplementary Figures 3, 7). The “ANME-1-related” group which is phylogenetically distinct from the canonical ANME-1 clade (Figure 5) was the most abundant ANME phylotype identified by *mcrA* sequencing (up to 97% at 875 cm core depth, Figure 3A). ANME-2a was the second-most abundant ANME phylotype (up to 60% at 575 cm core depth, Figure 3A). Quantitative PCR confirmed the abundance of ANME-1-related *mcrA* gene copies in the MZ (up to 3 × 10^5^ gene copies per gram wet sediment; Figure 4A). The distinct microbial communities of the three sites were visualized with Non-metric Multi-Dimensional Scaling (NMDS) plots, which indicated site-specific microbial community compositions (Figure 6 and Supplementary Figure 8). The site-wise clustering in NMDS was corroborated by PERMANOVA (*P* < 0.001) and by manyglm (*P* < 0.005), which both revealed a significant influence of the study site on the community structures. No significant correlation between pore-water profiles of the environmental parameters and individual members of the bacterial and archaeal communities could be found. Our multi-pattern indicator species analysis showed two different sets of typical microorganisms for the sulfidic sites, Church Trough and Royal Trough, and the iron-rich site Cumberland Bay fjord, respectively: ANME-1 was indicative for the sulfidic sites, whereas ANME-1-related was indicative for the iron-rich site (Supplementary Table 3).

## Discussion

Anaerobic oxidation of methane is an important filter mechanism for methane in marine sediments representing a major global methane sink (e.g., Niewöhner et al., 1998; Reeburgh, 2007; Knittel and Boetius, 2009). Generally, S-AOM is restricted to the SMT and performed by consortia of SRB and ANME (Boetius et al., 2000; Knittel and Boetius, 2009). However, ANME can still be present in MZ sediments below the SMT where they either perform S-AOM when residual sulfate is present (e.g., Treude et al., 2014) or utilize alternative electron acceptors such as Fe(III) oxides (e.g., Aromokeye et al., 2020). Recent studies indicate that ANME communities can be structured due to geochemical parameters such as methane flow intensity (Niemann et al., 2006) or sulfide concentrations (Roalkvam et al., 2011). Here, we present the dominant ANME subgroups in sediments of an iron-rich site located in a fjord setting (Cumberland Bay) in contrast to two sulfidic sites (Royal Trough and Church Trough) located on the outer Northern shelf of South Georgia.

### Electron Acceptor Availability Shapes the ANME Community Composition

The sediments of the northern shelf of South Georgia have previously been described as an area characterized by widespread active methane seepage, which has been attributed to high burial rates of organic matter fueling microbial methane production (Römer et al., 2014; Geprägs et al., 2016). The presence of high methane concentrations could be confirmed for the sediments at all sampling sites investigated here: Royal Trough and Cumberland Bay fjord were characterized by high methane concentrations and upward diffusive methane transport into the SMT (Figure 2A), whereas the core of Church Trough contained abundant methane hydrates below 120 cm core depth and represented a site of advective methane flux (Bohrmann et al., 2017). The main geochemical difference among the three sites, as detected by the pore-water profiles, is the availability of different electron acceptors.

The pore-waters retrieved at the Cumberland Bay fjord site contained abundant dissolved iron which largely consisted of Fe^2+^ (Figure 2B), a clear indication of a dominance of Fe(III) reduction over sulfate reduction; here, sulfate was below detection limit (<50 μM) below 425 cm core depth while sulfide was not detected at all (Figure 2A). However, residual sulfate concentrations were found below the SMT between 250 and 425 cm core depth (Figure 2A) which would be sufficient to enable a potential background sulfate reduction potentially coupled to AOM (Timmers et al., 2016) in addition to a potential Fe-AOM. The co-production of sulfide and ferrous iron as a result of concurrent Fe-AOM and S-AOM might explain the comparably low dissolved Fe pore-water concentrations in this zone. This is further supported by a kink in the pore-water methane concentration profile occurring between 533 and 633 cm core depth which coincides with a steep increase of dissolved Fe concentrations, indicating that Fe(III) reduction becomes a more important electron accepting process with depth compared to sulfate reduction (Figure 2B). Due to the fact that dissolved Fe and not sulfide was present at all depths, we suggest Fe(III) reduction as the primary electron accepting process in the MZ of Cumberland Bay sediments, whilst both Church Trough and Royal Trough are likely dominated by sulfate reduction due to high sulfate values accompanied by high sulfide concentrations (Figure 2A).

In the MZ of Church Trough and Royal Trough sediments the presence of sulfide below the SMT could either be a result of downward diffusion or indicate ongoing sulfate reduction (Figure 2A). Sulfate reduction in the MZ can occur at low rate, for example, when the sulfate pool is fueled by Fe(III)-driven re-oxidation of sulfide as observed in Aarhus Bay (Holmkvist et al., 2011) or sediments of the Nankai Trough (Riedinger et al., 2010). In the sediments of the sulfidic outer shelf sites of South Georgia, however, the concurrence of abundant sulfate and methane in the MZ allows for S-AOM, which may explain the high abundance of ANMEs (Figures 3, 4 and Supplementary Figures 4, 5). In Church Trough, where ANME represent up to 50% (at 122 cm core depth) of all archaeal 16S rRNA sequences (Supplementary Figure 4), S-AOM should be considered a dominant process. S-AOM in the MZ has been previously described for the Beaufort Sea, where simultaneous methanotrophic and sulfate-reducing activities were detected below the SMT and sulfate concentration was low (30–500 μM; Treude et al., 2014). Therefore, S-AOM is potentially not only an important metabolic process in the SMT but also in the MZ of both Royal Trough and Church Trough sediments.

The potential for AOM in Cumberland Bay fjord sediments (station PS81/284-1; GeoB22043-1) was previously demonstrated (Geprägs et al., 2016). Fe(III) reduction linked to AOM has been suggested to occur in the MZ of various iron-rich marine sediments (e.g., Riedinger et al., 2014; Egger et al., 2015, 2016a,b, 2017; Oni et al., 2015; Rooze et al., 2016; Aromokeye et al., 2020; Luo et al., 2020). In addition, Fe-AOM has been shown to contribute to the accumulation of dissolved iron in the pore-water of the MZ in North Sea sediments (Aromokeye et al., 2020). The presence of dissolved iron and absence of sulfide in the MZ of Cumberland Bay fjord sediments suggests that a potential AOM in the sediments below the SMT would be rather iron- than sulfate-dependent (Figure 2). AOM is generally performed by various subtypes of ANME (Boetius et al., 2000; Knittel and Boetius, 2009), therefore, we analyzed the microbial community by sequencing of the marker gene *mcrA* in addition to bacterial and archaeal 16S rRNA gene sequencing. In Cumberland Bay MZ sediment samples, ANME-1-related was the most abundant phylotype followed by ANME-2a, as revealed by sequencing of the *mcrA* gene (Figure 3A). ANME-1-related form a distinct clade (Figure 5) that appears to be rather versatile. It has recently been shown to grow in Fe-AOM performing sediment slurry incubations (Aromokeye et al., 2020), but it was also previously reported to be associated with S-AOM (Takeuchi et al., 2011). Despite this apparent versatility, ANME-1-related *mcrA* genes were far less abundant in the sulfidic sites with max. relative abundances of 1% (at 100 cm core depth and 312 cm core depth) in Church Trough and 15% (at 375 cm core depth) at Royal Trough (Figures 3B,C). ANME-2a were abundant in Cumberland Bay fjord with a max. relative abundance of 69% at 750 cm core depth (Figure 3A). This clade has been closely associated with Fe-AOM (Tu et al., 2017; Aromokeye et al., 2020) as well as S-AOM (Boetius et al., 2000) and is thought to be sensitive to high sulfide concentrations (Timmers et al., 2015).

In contrast, ANME-1 was the dominant ANME phylotype at both Church Trough and Royal Trough, followed by ANME-2c (Figures 3B,C). Both clades have been associated with S-AOM in multiple previous studies (e.g., Knittel et al., 2005; Roalkvam et al., 2011; Timmers et al., 2015) and have additionally been shown to tolerate high sulfate and sulfide concentrations (Timmers et al., 2015) which also characterize the deep sediments in Church Trough and Royal Trough. In addition, in an *in situ* community analysis, ANME-1 and ANME-2c have been shown to dominate the archaeal community in presence of high sulfide concentrations and close to gas hydrates (Roalkvam et al., 2011). The *mrcA* gene copy numbers of the respective ANME phylotypes were quantified with qPCR (Figure 4 and Supplementary Figure 6). In both Royal Trough and Church Trough, *mcrA* gene copies of the specific phylotypes co-occurred with *dsrA* gene copies over the entire core depth (Supplementary Figure 6). The concurrent presence of ANME-1 *mcrA* gene copies and *dsrA* gene copies could indicate a microbial potential for S-AOM that expands from the SMT into the MZ in both sulfidic sites of South Georgia. However, sediments from Cumberland Bay fjord also exhibit considerable quantities of *dsrA* gene copy numbers (Supplementary Figure 6). Potential *dsrA* harboring taxa, i.e., Desulfobulbaceae and Desulfobacterales, were abundant at all three sampling sites (Supplementary Figures 3–5). These comprise both Fe(III)- and sulfate-reducing (Lovley, 2013) organisms rendering the *dsrA* quantification inconclusive. The ANME-1 and ANME-2a/b/c phylotypes have been related to S-AOM in consortia with SRB in previous studies, though predominantly located in the SMT (Knittel and Boetius, 2009; Beulig et al., 2019). ANME-1 has been reported as the dominant archaeal group where they likely perform S-AOM in methane hydrate related biofilms in Arctic sediments (Gründger et al., 2019), and cold seep sediments (Vigneron et al., 2019). ANME-1 might perform methanogenesis based on phylogenetic assessments (Lloyd et al., 2011; Kevorkian et al., 2021), enrichments (Bertram et al., 2013), and their potential involvement in cryptic methane cycling in the SMT of Aarhus Bay sediment (Beulig et al., 2019). Given the high abundances of ANME-1 and near-absence of typical methanogens (e.g., *Methanosarcina* spp.) in the MZ of the sulfidic sites Royal Trough and Church Trough (Figure 3 and Supplementary Figures 4, 5), involvement of ANME-1 in both, methanogenesis and AOM is feasible. Nevertheless, other studies have demonstrated that methanogenesis during AOM occurs as an intrinsic back flux, but does not serve as an energy conserving reaction for ANME (Holler et al., 2011; Yoshinaga et al., 2014; Wegener et al., 2016). If this hypothesis holds true, ANME-1 in Royal Trough and Church Trough sediments are more likely to perform S-AOM which is supported by the presence of sulfate and sulfide in the pore-water (Figure 2). Consequently, methanogenesis as an energy conserving reaction would be restricted to deeper sediment layers (i.e., below 10 m core depth) and supposedly be performed by known methanogens.

Analyses of the β-diversities of the operational taxonomic units (OTU) generated by sequencing of the bacterial and archaeal 16S rRNA gene as well as the *mcrA* gene revealed the site-specific influence on the community composition (Figure 6). The ANME community of the sulfidic sites Church Trough and Royal Trough were dominated by ANME-1 and ANME-2c (Figures 3B,C, 4B,C and Supplementary Table 3) in contrast to the iron-rich site Cumberland Bay, where ANME-1-related and ANME-2a were the most abundant ANME phylotypes (Figures 3A, 4A and Supplementary Table 3). Thus, it is highly likely that the electron acceptor availability might heavily impact ANME community composition.

### ANME-1-Related and ANME-2a Mediate Potential Fe-AOM in Cumberland Bay

In the sediments at the Cumberland Bay fjord site, potential Fe-AOM might be mediated by ANME-1-related or ANME-2a, or in consortia with bacterial iron reducers, e.g., members of the Desulfuromonadales as indicated previously (Oni and Friedrich, 2017; Aromokeye et al., 2020). Nevertheless, the Desulfuromonadales as potential bacterial partners in Fe-AOM were found at low abundance (<2%) at all sites (Supplementary Figure 3) only. Other potential bacterial partners might be members of the Desulfobacterales, which are also known to use ferric iron as electron acceptor (Lovley, 2013), albeit their relative abundance was similar across sites. A deeper characterization of the physiological role of ANME-2a requires enrichment (or pure) cultures, which are however, notoriously difficult to obtain. Further investigations would therefore be required to determine the true potential of Fe(III) reducing bacteria in South Georgia. ANME-2a are closely related to Methanoperendenaceae (or ANME-2d; Figure 5) of which many members have been shown to mediate Fe-AOM or Mn-AOM (Ettwig et al., 2016; Cai et al., 2018; Shen et al., 2019; Leu et al., 2020). Generally, Fe-AOM associated ANME phylotypes such as *Candidatus* “*Methanoperedens nitroreducens*” (Haroon et al., 2013; Ettwig et al., 2016) and *Candidatus* “*Methanoperedens ferrireducens*” of the family Methanoperendenaceae (Cai et al., 2018), the ANME-2a group (Wang et al., 2014) or the ANME-1 group (Meyerdierks et al., 2010) and Mn-AOM associated ANME phylotypes such as *Candidatus* “*Methanoperedens manganicus*” and *Candidatus* “*Methanoperedens manganireducens*” (Leu et al., 2020) possess several multi-heme cytochrome encoding genes typically associated with dissimilatory Fe(III) and Mn(IV) reduction in bacteria (Lovley, 2013; McGlynn et al., 2015; Wegener et al., 2015; Vigneron et al., 2019). The ANME-1-related archaea form a distinct order-level lineage apart from ANME-1 (Figure 5; Takeuchi et al., 2011; Lever and Teske, 2015). So far, their metabolism remains largely elusive; Takeuchi et al. (2011) concluded that ANME-1-related archaea mediate S-AOM, although the addition of sulfate did not stimulate AOM activity in all their incubations in which ANME-1-related archaea were the most abundant ANME group. However, in MZ sediments from the North Sea ANME-1-related cell numbers strongly correlated with the dissolved iron pore-water profile (Aromokeye et al., 2020). In our study, we could not find such a correlation of pore-water iron concentration and ANME-1-related *mcrA* gene copy numbers. Nonetheless, ANME-1-related and ANME-2a OTUs were characteristic for the sediments at the Cumberland Bay fjord site as determined by multi-pattern indicator species analysis (Supplementary Table 3). In contrast, ANME-1 and ANME-2c OTUs were indicative for the sulfidic sites, Royal Trough and Church Trough (Supplementary Table 3).

The abundance of potentially Fe-AOM mediating ANME phylotypes such as ANME-1-related and ANME-2a coincides with a potential for AOM in MZ sediments of Cumberland Bay fjord (Geprägs et al., 2016). In our analyses of the pore-water constituents, however, we detected dissolved iron but no sulfide at this depth (Figure 2). This demonstrates a dominance of iron reduction over sulfate reduction in the MZ of Cumberland Bay sediments suggesting that iron reduction below the SMT may be either fueled by methane or organic matter oxidation. Since ANME-1-related and ANME-2a are the most dominant ANME phylotypes, they are the most promising candidates for potential Fe-AOM in the MZ sediments of the Cumberland Bay fjord site.

## Conclusion

The methane-abundant and methane seepage-associated sediments on the northern shelf of South Georgia Island display a wide array of ANME subgroups in varying abundance. We find that ANME communities are likely influenced by the availability of different electron acceptors. ANME-1-related and ANME-2a are the most abundant ANME groups in the iron-rich sediments of the Cumberland Bay fjord site, whereas ANME-1 and ANME-2c are the most abundant ANME groups in the sulfidic sediments of the outer shelf sites Church Trough and Royal Trough. Although we do not exclude the possible impact of other selective factors such as community competition and dispersal, our study indicates that the electron acceptor availability has a strong selective effect on the ANME community in South Georgia sediments.

## Materials and Methods

### Sampling Sites and Sampling Procedure

The northern shelf of South Georgia Island, Scotia Sea, South Atlantic Ocean, is characterized by widespread occurrence of active methane seepage (Römer et al., 2014; Geprägs et al., 2016). Sediment and pore-water samples were taken from three sampling locations around South Georgia by means of gravity coring: GeoB22032-1 (Church Trough, water depth: 369.0 m), GeoB22039-2 (Royal Trough, water depth: 227.0 m) and GeoB22043-1 (Cumberland Bay fjord, water depth: 254.0 m) sampled during the R/V METEOR cruise M134 in 2017 and PS81/284-1 (Cumberland Bay fjord, water depth: 274.6 m) during RV POLARSTERN cruise PS81 in 2013 (Figure 1 and Supplementary Table 1). The geology and geochemistry of the northern shelf and fjords were extensively studied during these cruises (Bohrmann, 2013; Bohrmann et al., 2017). All GC positions are characterized by their location within a trough and their proximity to an active methane seep site. The cores GeoB22039-2 from Royal Trough and GeoB22032-1 from Church Trough have previously been characterized as sites with high sulfide concentrations (Bohrmann, 2013). In addition, GC GeoB22032-1 from Church Trough was shown to contain methane hydrates and methane at saturation, whereas the sediments at the site in Royal Trough had only dissolved methane in the MZ.

Pore-water samples were retrieved by filtering through rhizon samplers (average pore size 0.15 μm; Seeberg-Elverfeldt et al., 2005) and sediment samples for CH_4_ analyses were taken as described in Pape et al. (2014). Concentrations of pore-water constituents of core GeoB22032-1 (Church Trough) are likely somewhat diluted due to the dissolution of the gas hydrates during core retrieval. Sediment samples for DNA extraction were taken in a sterile manner and stored at −20°C immediately as described in Aromokeye et al. (2020).

### Geochemical Analyses

Measurements of pore-water sulfate (detection limit of 50 μM) and sulfide were performed as described by Oni et al. (2015). CH_4_ measurements were performed as described by Pape et al. (2014). DIC was measured with a flow injection system according to Hall and Aller (1992). Dissolved iron and manganese were measured with *inductively coupled plasma optical emission spectrometry* (ICP-OES) as described in Aromokeye et al. (2020). In addition, pore-water Fe^2+^ concentrations were determined with a Ferrozine based photometric essay directly after retrieval of pore-water onboard. 1 ml pore-water aliquots were transferred into cuvettes pre-filled with 50 μl of Ferrozine solution immediately after pore-water retrieval on board. Fe^2+^ concentrations were measured photometrically at a wavelength of 565 nm.

### Nucleic Acid Extraction

DNA was extracted from 0.5 g of sediment per depth in technical triplicates with the phenol-chloroform-isoamyl alcohol-method (Lueders et al., 2004). The DNA in the triplicates was pooled during elution with 50 μl diethyl pyrocarbonate (DEPC) treated water and stored at −20°C until further use. Single extracts per sampling depth were used for subsequent sequencing and qPCR analyses.

### Illumina Sequencing of 16S rRNA Genes

Illumina of 16S rRNA genes was performed as described previously (Aromokeye et al., 2020). Briefly, amplification was performed using KAPA HiFi DNA polymerase (KAPA Biosystems, Germany) with barcoded versions of the primer pair Bac515F (5′-GTGYCAGCMGCCGCGGTAA-3′) (Parada et al., 2016) and Bac805R (5′-GACTACHVGGGTATCTAATCC-3′) (Herlemann et al., 2011) for targeting bacteria, and barcoded versions of the primer pair Arc519F (5′-CAGCMGCCGCGGTAA-3′) (Ovreås et al., 1997) and Arc806R (5′-GGACTACVSGGGTATCTAAT-3′) (Takai and Horikoshi, 2000) were used for targeting archaea. Purified and quantified PCR products were sequenced on an Illumina Sequencing HiSeq 4000 (2 × 150 bp) platform (GATC Biotech GmbH, Germany) and analyzed using SILVA database v.1.32 (Quast et al., 2012) in a QIIME environment (Caporaso et al., 2010, 2011).

### Illumina Sequencing of *mcrA* Genes

Illumina of *mcrA* genes was performed as described previously (Aromokeye et al., 2020). Briefly, amplification was performed using ALLin RPH (high Qu) polymerase kit (Thermo Fisher Scientific, Germany) with barcoded versions of the primer pair mlasF (5′-GGTGGTGTMGGDTTCACMCARTA-3′, Steinberg and Regan, 2008) and ME2mod (5′-TCATBGCRTAGTTNGGRTAGT-3′, Mori et al., 2012). Purified and quantified PCR products were analyzed on an Illumina Sequencing MiSeq (2 × 300 bp) platform (MR DNA, Molecular Research LP, Shallowater, TX, United States).

### Quantitative PCR of Bacterial and Archaeal 16S rRNA Genes, *dsrA* and Total *mcrA* Genes

Quantitative PCR of bacterial and archaeal 16S rRNA genes was performed using MESA BLUE qPCR MasterMix Plus for SYBR^®^ Assay Low ROX (Eurogentec, Seraing, Belgium) and the respective primer pairs (Supplementary Table 2). DNA extracted from *in situ* sediment samples was quantified using Quant-iT PicoGreen dsDNA assay kit (Invitrogen-Thermo Fischer Scientific, Steinheim, Germany) and diluted to 0.5 ng/μL. 2 μL of diluted DNA was used as template for all qPCR assays. qPCR assays were run using the following program: 95°C: 10 min; 40 cycles at 95°C: 30 s, 58°C or 60°C: 20 or 30 s, 72°C: 40 s. A post amplification melting curve analysis was performed in order to confirm the absence of PCR by-products by detecting change in fluorescence every 0.5°C from 60 to 95°C. Detailed information on qPCR primers, assay conditions, efficiencies and standards is provided in Supplementary Table 2.

### Quantification of *mcrA* Genes

Quantitative PCR of *mcrA* genes was performed as described previously (Aromokeye et al., 2020). Detailed information on qPCR primers, assay conditions, efficiencies and standards is provided in Supplementary Table 2.

### Analysis of 16S rRNA and *mcrA* Gene Sequences

Sequence analysis was performed on the QIIME 1.9 platform (Caporaso et al., 2010) based on the analysis pipeline as recommended (Pylro et al., 2014) with modifications. To analyze *mcrA* gene sequences, barcodes were extracted and sequences were reoriented starting with the forward primer sequence. Reoriented reads were joined using a minimum overlap of 50 bases. Joined reads were demultiplexed with a filter quality of Q0 (Caporaso et al., 2011). Demultiplexed sequences were quality filtered using USEARCH 11 (expected error value of 0.5) (Edgar, 2010). At this step, all sequences were truncated to a length of 352 bp. USEARCH 11 was further used to dereplicate sequences, sort them by their abundances and subject them to remove singletons. OTU clustering and chimera removal was done using the UPARSE-OTU algorithm (Edgar, 2013) to create an OTU database. Chimeric sequences were checked and discarded by the UPARSE-OTU algorithm during this step. The truncated, non-dereplicated reads were mapped back to the OTU database to create an OTU table. OTUs were classified for their taxonomy using uclust and an in-house *mcrA* gene database which was created by acquisition of long (>1000 bp) gene sequences of cultured and published methanogenic, methanotrophic and hydrocarbon degrading archaea (from https://www.ncbi.nlm.nih.gov/nucleotide/) that were manually aligned in ARB 6.02 (Ludwig et al., 2004) as described in Aromokeye et al. (2020). The taxonomic assignment was done on the family level at a sequence identity of 0.7 (Yang et al., 2014). Using the RAxML algorithm in ARB, a phylogenetic tree was constructed, to which shorter mcrA gene sequences were added using the ARB Parsimony tool. The OTU table and taxonomy assignment files were merged together using a set of “biom” commands (McDonald et al., 2011) to obtain a tab-delimited text file useful for downstream analysis. A few modifications of the above pipeline were done to analyze 16S rRNA gene sequences. Only forward reads were used to analyze the community composition. After extraction of barcodes, forward reads were de-multiplexed, quality filtered and their lengths were truncated to 143 bp. Taxonomic assignment was done on clustered OTUs against the 16S rRNA gene SILVA database (Release 132 for QIIME) (Quast et al., 2012).

### Statistical Analysis

Analyses and figures (except Figure 5) were made within the R environment v. 3.3.2 (R Core Team, 2019). Community analyses were performed with the package vegan (Oksanen et al., 2018). The variation of microbiological communities between the three GCs based on bacterial 16S rRNA gene, archaeal 16S rRNA gene and *mcrA* gene sequencing was visualized with Non-metric Multi-Dimensional Scaling (NMDS) based on Bray-Curtis dissimilarities. To further elucidate the association between ANME organisms and our three study sites (individually and in combination), we carried out a multipattern indicator species analysis using the **multipatt** function in the indicspecies package under default parameters with 999 permutations (De Cáceres and Legendre, 2009). We conducted PERMANOVA with Bray Curtis distances under 999 permutations in vegan (function “adonis2”; Oksanen et al., 2018) to account for the influence of the study site (Cumberland Bay, Church Trough, and Royal Trough) on the community structure. In addition, we used the function ‘manyglm’ (package mvabund; Wang et al., 2012) with 300 Monte-Carlo permutations and the Wald test to account for the influence of the study site on the community structure.

## Data Availability Statement

Raw sequence data used in this study can be accessed from Sequence Read Archive with the bioproject accession number PRJNA658241. Core descriptions and relevant geochemical data are made available through the World Data Center PANGAEA^®^ (www.pangaea.de).

## Author Contributions

AS, DA, SB, PR, SK, and MF designed the study. DA, ID, TP, and SK performed the geochemical sampling and analysis. AS, AK, LW, and LM performed the molecular biology assessments. AS and AK performed the phylogenetic analysis with a data base built by AK. AS performed the figures production. TR-H performed the statistical analysis. GB and SK developed the cruise design. GB, SK, and MF obtained funding for this research. AS together with MF wrote the manuscript with support from all co-authors. All authors contributed to the article and approved the submitted version.

## Conflict of Interest

The authors declare that the research was conducted in the absence of any commercial or financial relationships that could be construed as a potential conflict of interest.
