# Supplementary material for: Electron Acceptor Availability Shapes Anaerobically Methane Oxidizing Archaea (ANME) Communities in South Georgia Sediments
Source: Front Microbiol. 2021 Apr 14;12:617280. doi: 10.3389/fmicb.2021.617280 (PMC8081031; doi:10.3389/fmicb.2021.617280)
Supplement: Supplementary file 1 [file Table_1.docx]

Supplementary Material

**Supplementary Methods**

**Supplementary Table 1.** Sampling information for all gravity cores retrieved from South Georgia during the research cruises PS81 with RV POLARSTERN in 2013 and M134 with RV METEOR in 2017.

| Location | Station name | Water depth | Coordinates | |
| --- | --- | --- | --- | --- |
|  |  |  | **Latitude** | **Longitude** |
| Church Trough | GeoB22032-1 | 369 m | 53°46.199’S | 38°8.408’W |
| Cumberland Bay | GeoB22043-1 | 254 m | 54°15.899’S | 36°26.248’W |
| Royal Trough | GeoB22039-2 | 227 m | 54°27.438’S | 35° 50.54’W |
| Cumberland Bay | PS81/284-1 | 259 m | 54°15.910’S | 36°26.230’W |

**Supplementary Table 2.** qPCR conditions and primers.

| Target gene | Annealing temperature/time | Average efficiency | R^2^ | Template | Mass of  one gene (Da) | Primer sequences (5' - 3') | Reference | Primer  concentration | Product  length (bp) |
| --- | --- | --- | --- | --- | --- | --- | --- | --- | --- |
| Bacterial 16S rRNA | 58 °C/  20 seconds | 74.5% | >0.99 | *E.coli* SB1 | 928817 | F: AGAGTTTGATYMTGGCTCAG  R: GCWGCCWCCCGTAGGWG | (Eden et al. 1991)  (Yu et al., 2005) | 300 nM | 250 bp |
| Archaeal 16S rRNA | 58 °C/  20 seconds | 102.1% | >0.99 | *Methanosarcina*  *barkeri* DSM800 | 825572 | F: ATTAGATACCCSBGTAGTCC  R: GTGCTCCCCCGCCAATTCCTTTA | ( modified from Yu et al., 2005)  (modified from Lueders and Friedrich, 2002) | 300 nM | 250 bp |
| *dsrA* | 60 °C/  30 seconds | 95.5% | >0.99 | *Desulfovibrio burkiniensis* DSM6830 | 1184711 | F: ACSCACTGGAAGCACGGCGG  R: GTGGMRCCGTGCAKRTTGG | (Kondo et al., 2004) | 400 nM | 221 bp |
| General *mcrA* | 58 °C/  30 seconds | 80.7% | >0.99 | A4-67 | 898620 | F: GTCNGGTGGHGTMGGSTTYAC  R: TCATBGCRTAGTTNGGRTAGT | (Nunoura et al. 2008)  (Mori et al. 2012) | 600 nM | 475 bp |
| ANME-1 *mcrA* | 62 °C/  30 seconds | 101.4% | >0.99 | E-3 | 878414 | F: AYGACCAGYTGTGGTTCGGAACGT  R: TCCATGTTSARCTTGTCGCCCTTY | (Miyazaki et al., 2009) | 600 nM | 175 bp |
| ANME-2a *mcrA* | 62 °C/  30 seconds | 85% | >0.99 | F-79 | 862362 | F: ATATGGCAGATATTGTCCAGACCTCAAGG  R: ATTTATCCCAKCCGTAYTC |  | 600 nM | 218 bp |
| ANME-2c *mcrA* | 59 °C/  20 seconds | 92.7% | >0.99 | E-79 | 928817 | F: AAGGAYATYRSAACCGAATC  R: TTGAAAGGTACCATSSKGAAAGACC |  | 600 nM | 180 bp |
| ANME-1- related *mcrA* | 52 °C/  30 seconds | 89.1% | >0.99 | E-155 | 870491 | F: GAGATCGCVRTVGACATGTTCGG  R: GCCCTMACAGAMCCRCCGAAGTG | (Zhou et al., 2014) | 400 nM | 172 bp |

**Supplementary Results**


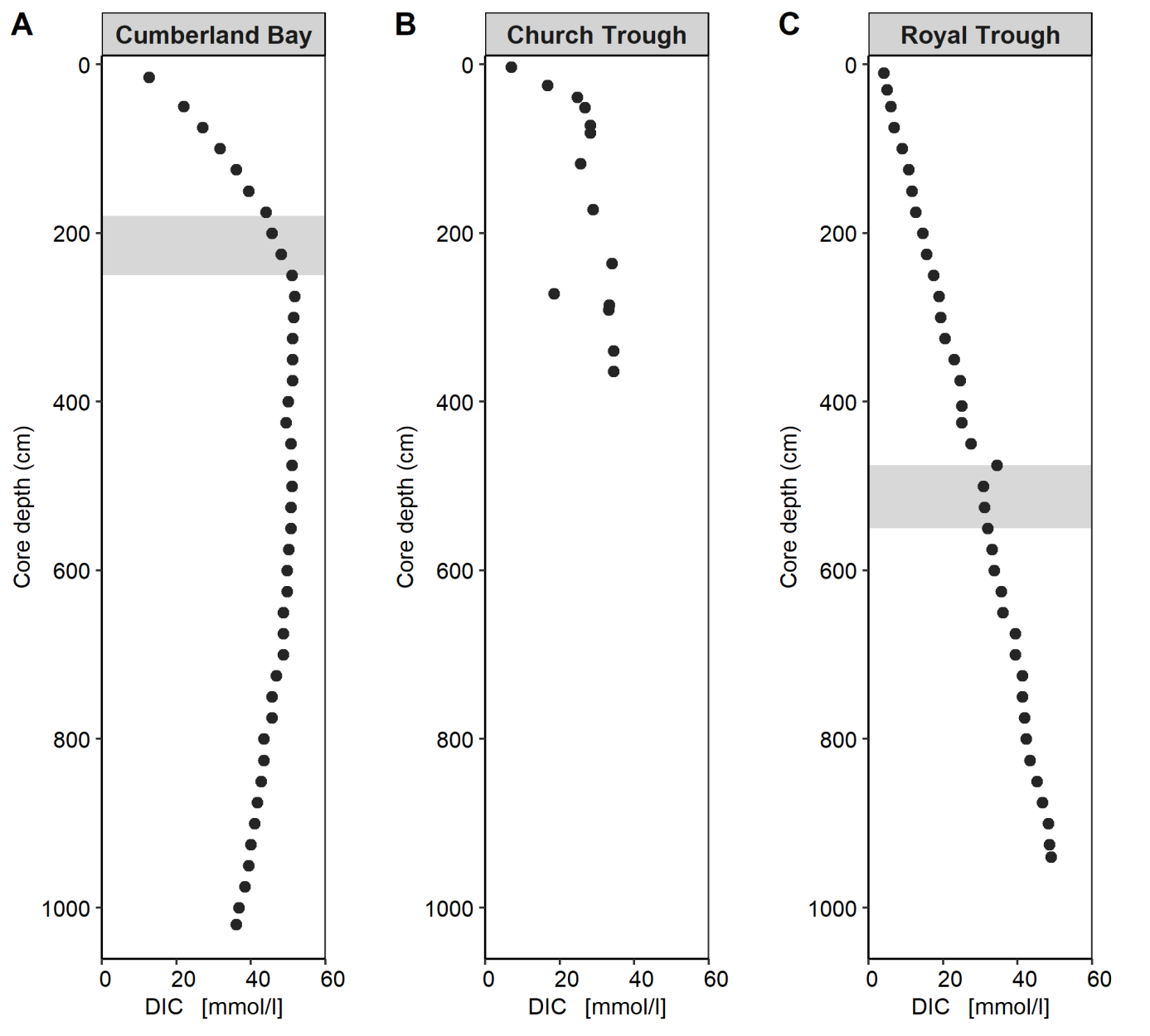


**Supplementary Figure 1:** Concentration of dissolved inorganic carbon (DIC) in the pore-waters of Cumberland Bay, Church Trough, and Royal Trough. Grey bars indicate depth of the SMT.


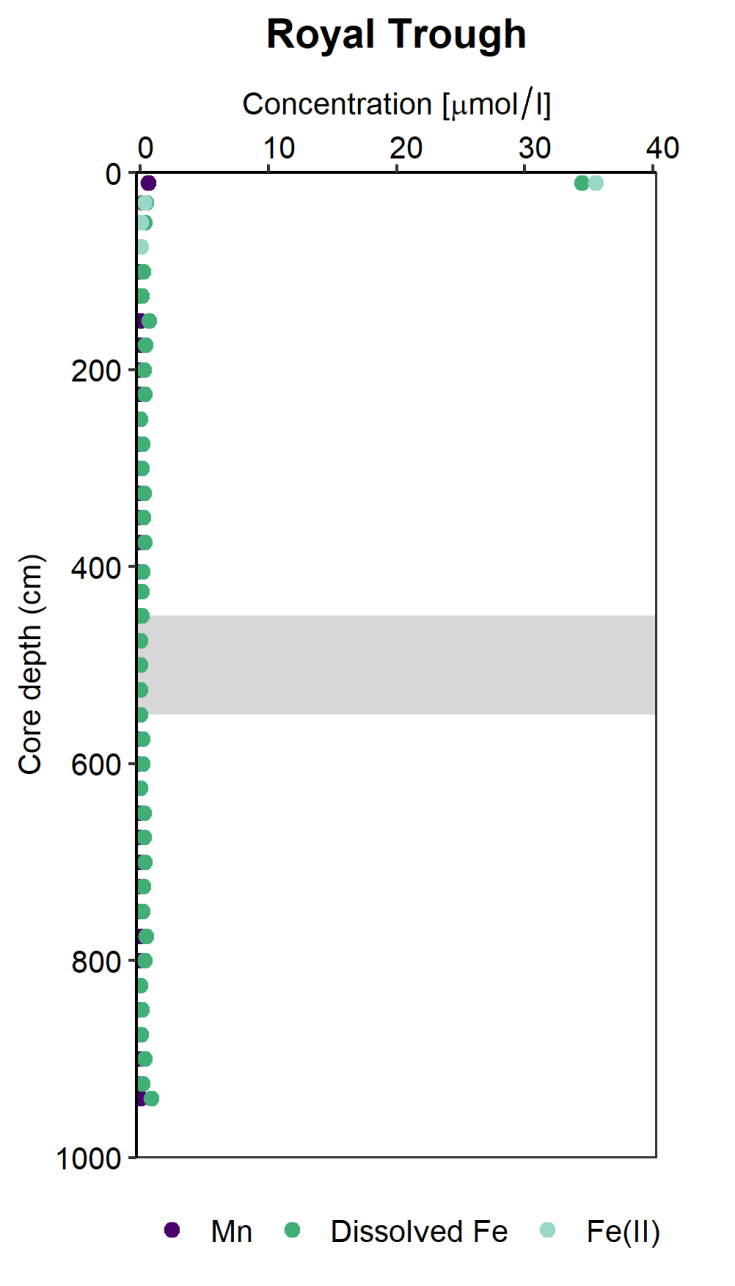


**Supplementary Figure 2:** Concentration of dissolved manganese and iron in the pore-water of Royal Trough. Grey bars indicate depth of the SMT.

**
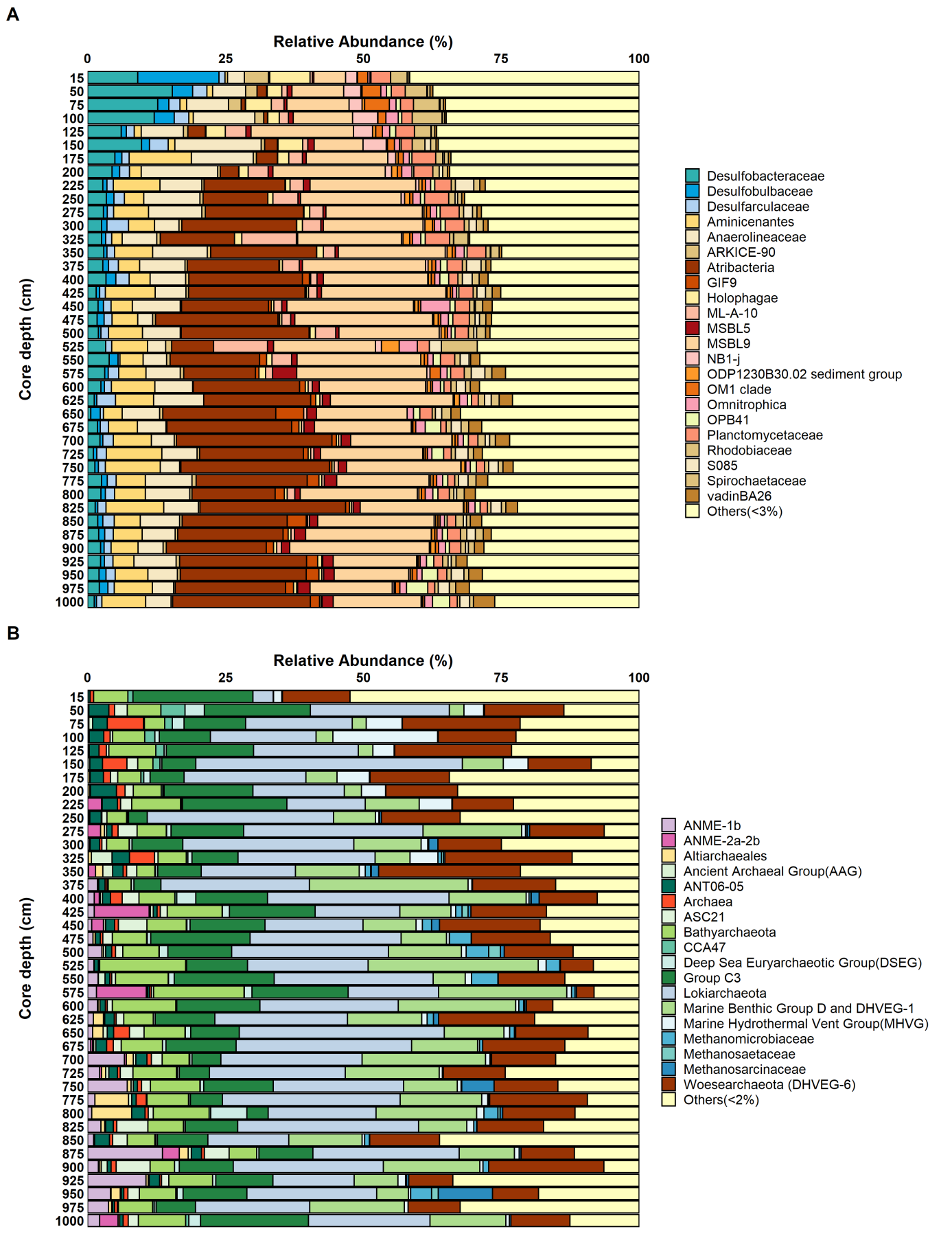
 Supplementary Figure 3:** Total sum scaling of bacterial **(A)** and archaeal **(B)** 16S rRNA genes over core depth in sediments at station GeoB22043-1 (Cumberland Bay).


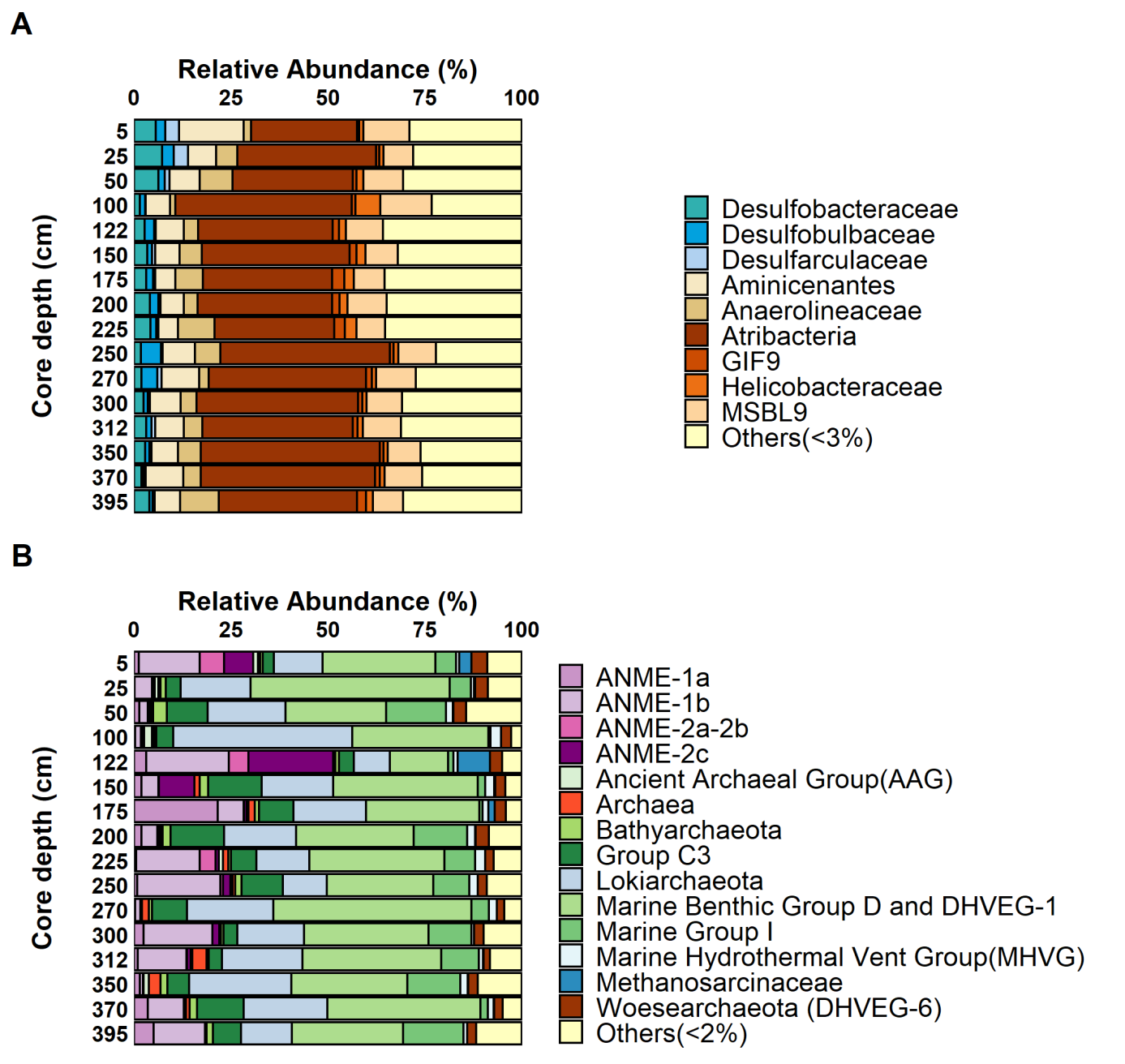


**Supplementary Figure 4:** Total sum scaling of bacterial **(A)** and archaeal **(B)** 16S rRNA genes over core depth in sediments at station GeoB22032-1 (Church Trough).

**
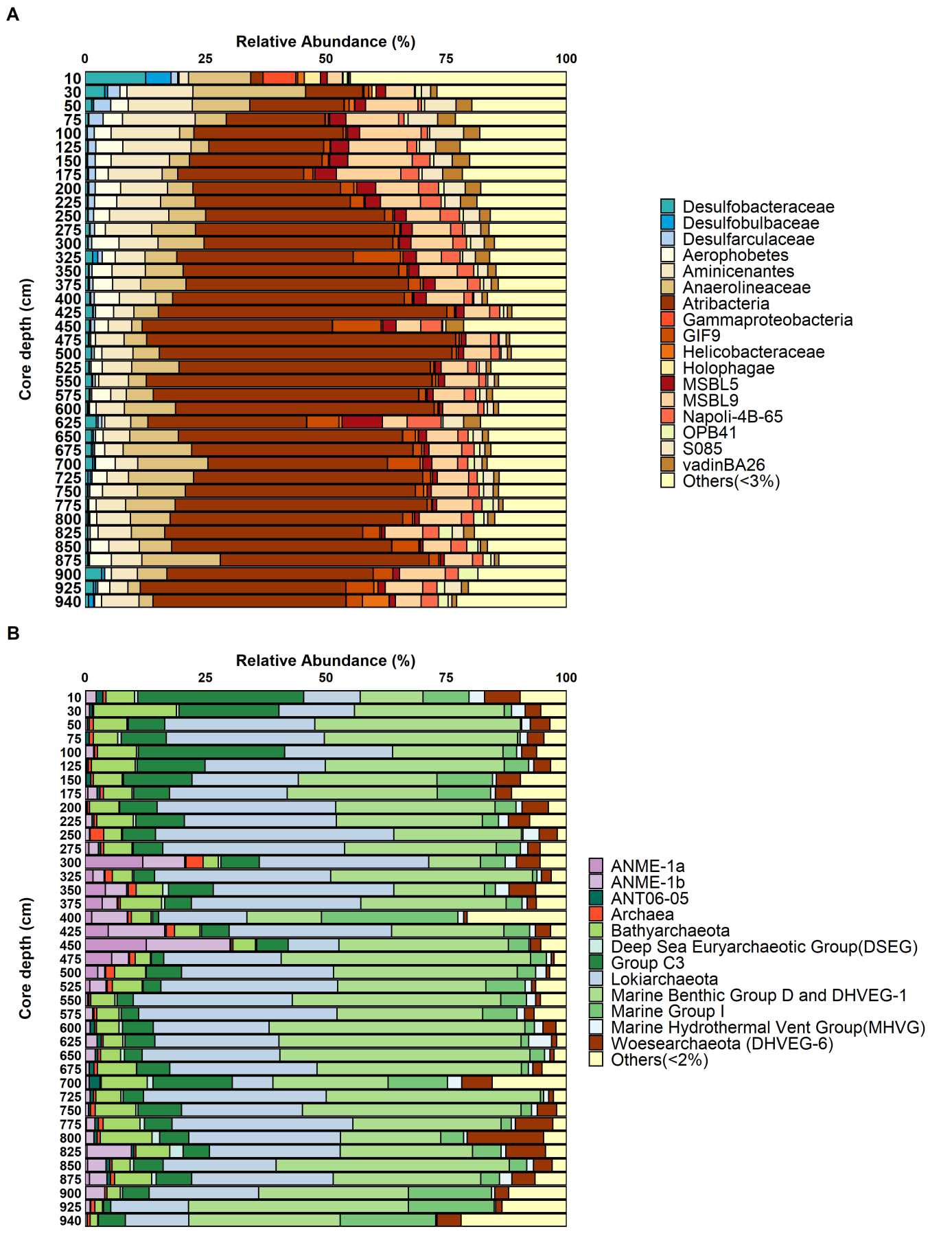
 Supplementary Figure 5:** Total sum scaling of bacterial **(A)** and archaeal **(B)** 16S rRNA genes over core depth in sediments at station GeoB22039-2 (Royal Trough).


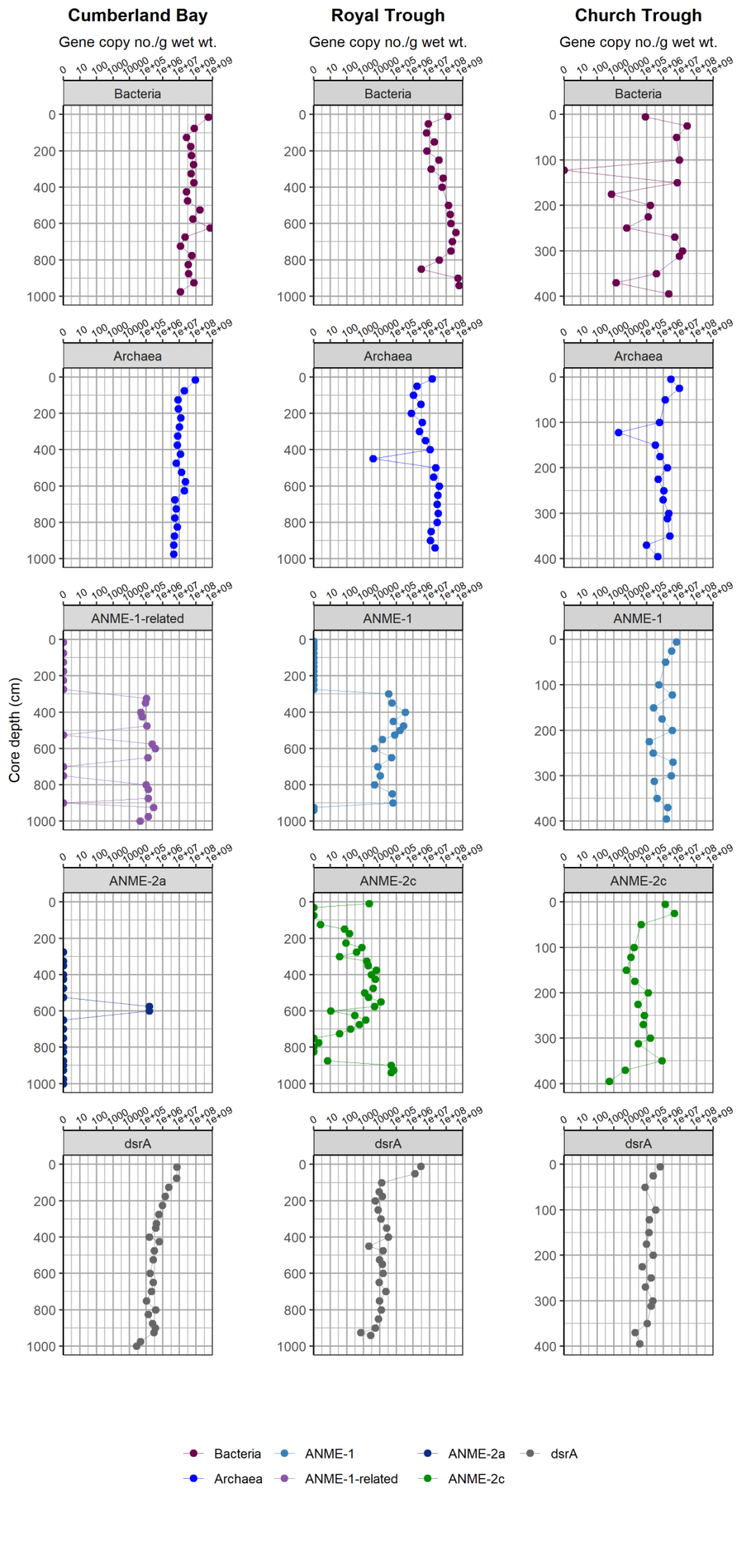


**Supplementary Figure 6:** Gene copy numbers of bacterial and archaeal 16S rRNA, *dsrA*, general *mcrA* and specific ANME groups (*mcrA* gene-based). ANME-1-related and ANME-2a *mcrA* gene copies were quantified for sediments at **(A)** Cumberland Bay, ANME-1 and ANME-2c *mcrA* gene copies were quantified for **(B)** Royal Trough and **(C)** Church Trough.


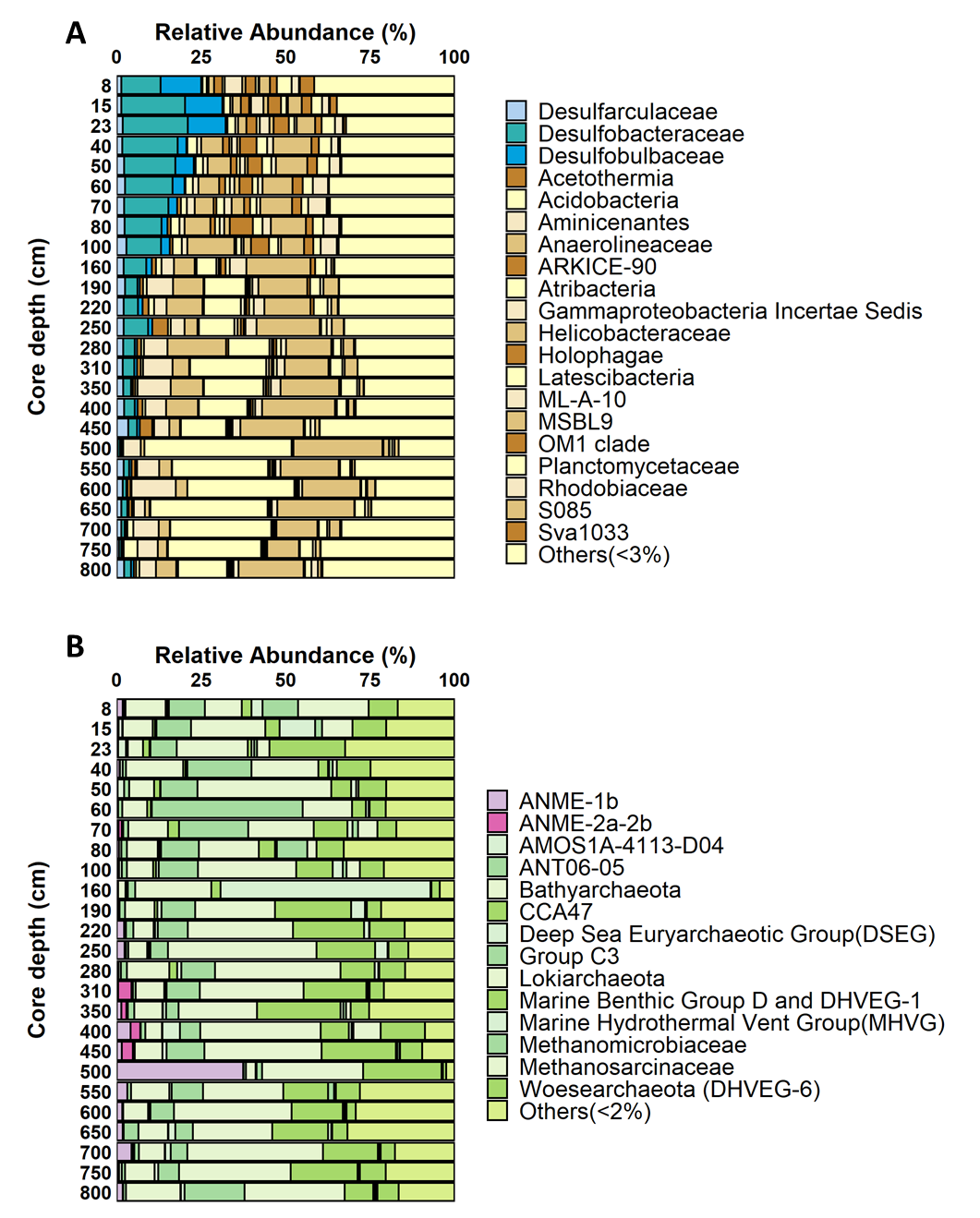


**Supplementary Figure 7:** Total sum scaling of bacterial **(A)** and archaeal **(B)** 16S rRNA genes over core depth in sediments at station PS81/284-1 (Cumberland Bay).


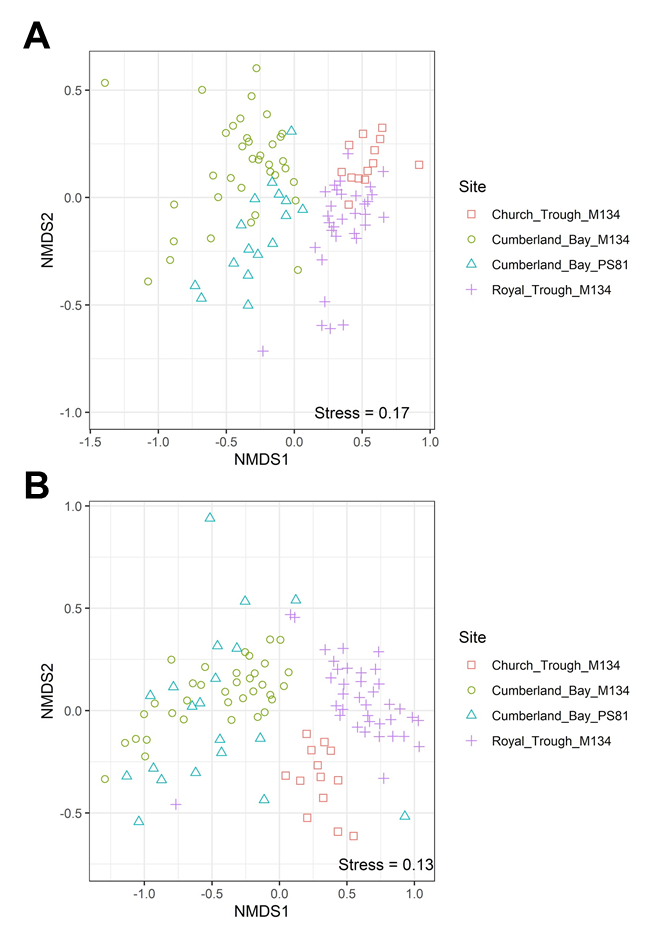


**Supplementary Figure 8:** Site-specific β-diversity patterns of sediments at sampling sites in Church Trough, Royal Trough and Cumberland Bay. NMDS ordination of abundant OTUs (> 1000 sequences, pooled from all sampling depths) based on sequencing of the **(A)** bacterial 16S rRNA gene and **(B)** archaeal 16S rRNA gene.

Supplementary Table 3. Multi-pattern indicator species analysis of the iron-rich fjord site Cumberland Bay (CB) v. the sulfidic outer shelf sites Church Trough and Royal Trough (CT+RT).

| **Site** | **OTU** | **stat** | **p.value** | **Phylogenetic group** |
| --- | --- | --- | --- | --- |
| CB | OTU7250875233646 | 0.982 | 0.001 | ANME-1_related |
| CB | OTU4993752709982 | 0.947 | 0.001 | ANME-1_related |
| CB | OTU58392867070580 | 0.946 | 0.018 | ANME-1_related |
| CB | OTU99609141860592 | 0.943 | 0.001 | ANME-1_related |
| CB | OTU14028907976675 | 0.916 | 0.001 | ANME-1_related |
| CB | OTU22842546051745 | 0.904 | 0.027 | ANME-1_related |
| CB | OTU76001742019632 | 0.849 | 0.001 | ANME-1_related |
| CB | OTU44237092451895 | 0.81 | 0.003 | ANME-1_related |
| CB | OTU55742385971646 | 0.79 | 0.006 | ANME-2a |
| CB | OTU36302135709074 | 0.785 | 0.001 | ANME-1_related |
| CB | OTU96108113213176 | 0.757 | 0.012 | ANME-1_related |
| CB | OTU17253412194672 | 0.755 | 0.015 | ANME-2a |
| CB | OTU94733049239730 | 0.729 | 0.029 | ANME-2a |
| CB | OTU1425746307231 | 0.701 | 0.011 | ANME-3 |
| CB | OTU79621886658538 | 0.693 | 0.007 | ANME-1_related |
| CB | OTU75824173950467 | 0.688 | 0.012 | ANME-1_related |
| CB | OTU2357083689555 | 0.67 | 0.005 | ANME-2a |
| CB | OTU32629470417825 | 0.669 | 0.021 | ANME-1_related |
| CB | OTU50629696844004 | 0.647 | 0.014 | ANME-1_related |
| CB | OTU22094956756012 | 0.636 | 0.035 | ANME-2a |
| CB | OTU48075336525311 | 0.617 | 0.024 | ANME-1_related |
| CB | OTU90520993835481 | 0.596 | 0.028 | ANME-3 |
| CB | OTU88069379059672 | 0.55 | 0.011 | ANME-1_related |
| CB | OTU33809472464228 | 0.548 | 0.014 | ANME-2a |
| CB | OTU52337634963561 | 0.547 | 0.026 | ANME-1_related |
| CB | OTU25628120130388 | 0.546 | 0.013 | ANME-1_related |
| CB | OTU85813604236348 | 0.521 | 0.029 | ANME-2 related |
| CB | OTU7181963320866 | 0.426 | 0.025 | ANME-1_related |
| CT+RT | OTU11871138437443 | 0.998 | 0.001 | ANME-1 |
| CT+RT | OTU95648754948505 | 0.99 | 0.001 | ANME-1 |
| CT+RT | OTU50994400614048 | 0.984 | 0.008 | ANME-2c |
| CT+RT | OTU4907547411868 | 0.972 | 0.001 | ANME-1 |
| CT+RT | OTU58053388386188 | 0.959 | 0.001 | ANME-1 |
| CT+RT | OTU80493260073156 | 0.959 | 0.001 | ANME-1 |
| CT+RT | OTU48102003243571 | 0.954 | 0.001 | ANME-1 |
| CT+RT | OTU36428424728318 | 0.943 | 0.001 | ANME-1 |
| CT+RT | OTU48029910303795 | 0.94 | 0.001 | ANME-1 |
| CT+RT | OTU92301110519456 | 0.92 | 0.001 | ANME-1 |
| CT+RT | OTU59177098849705 | 0.917 | 0.003 | ANME-1 |
| CT+RT | OTU67467151390469 | 0.905 | 0.001 | ANME-1 |
| CT+RT | OTU89175956232636 | 0.896 | 0.001 | ANME-1 |
| CT+RT | OTU81790005087083 | 0.892 | 0.005 | ANME-1 |
| CT+RT | OTU51704145863855 | 0.877 | 0.001 | ANME-1 |
| CT+RT | OTU8506791972073 | 0.857 | 0.001 | ANME-1 |
| CT+RT | OTU56469656389629 | 0.834 | 0.001 | ANME-1 |
| CT+RT | OTU27114013796413 | 0.785 | 0.004 | ANME-1 |
| CT+RT | OTU78189899217201 | 0.523 | 0.026 | ANME-1 |
| CT+RT | OTU72065222373213 | 0.474 | 0.022 | ANME-1 |

**Supplementary References**

Eden, P.A., Schmidt, T.M., Blakemore, R.P., and R. Pace, N.R. (1991). Phylogenetic analysis of *Aquaspirillum magnetotacticum* using polymerase chain reaction-amplified 16S rRNA-specific DNA. Inter. Journ. Sys. Evol. Microbiol*.* 4, 324-325. doi: 10.1099/00207713-41-2-324.

Kondo, R., Nedwell, D.B., Purdy, K. J., Silva and S.Q. (2004). Detection and enumeration of sulphate-reducing bacteria in estuarine sediments by competitive PCR. Geomicrobiol. *Journ.* 21, 145-157. Lueders, T., and Friedrich, M.W. (2002). Effects of amendment with ferrihydrite and gypsum on the structure and activity of methanogenic populations in rice field soil. Appl. Environ. Microbio*l*. 68, 2484-2494. doi: 10.1128/AEM.68.5.2484-2494.2002.

Miyazaki, J., Higa, R., Toki, T., Ashi, J., Tsunogai, U., Nunoura, T., Imachi, H., and Takai, K. (2009). Molecular characterization of potential nitrogen fixation by anaerobic methane-oxidizing archaea in the methane seep sediments at the number 8 Kumano Knoll in the Kumano Basin, offshore of Japan. Appl. Environ. Microbiol*.* 75**,** 7153-7162. doi: 10.1128/aem.01184-09.

Mori, K., Iino, T., Suzuki, K.-I., Yamaguchi, K., and Kamagata, Y. (2012). Aceticlastic and NaCl-requiring methanogen “*Methanosaeta pelagica*” sp. nov., isolated from marine tidal flat sediment. Appl. Environ. Microbiol*.* 78**,** 3416-3423. doi: 10.1128/aem.07484-11.

Nunoura, T., H. Oida, J. Miyazaki, A. Miyashita, H. Imachi, and Takai, K. (2008). Quantification of *mcrA* by fluorescent PCR in methanogenic and methanotrophic microbial communities. FEMS Microbiol. Ecol. 64, 240-247. doi: 10.1111/j.1574-6941.2008.00451.x.

Yu, Y., Lee, C., Kim, J. and Hwang, S. (2005). Group-specific primer and probe sets to detect methanogenic communities using quantitative real-time polymerase chain reaction. Biotechnol. Bioengin. 89, 670-679. doi: 10.1002/bit.20347.

Zhou, Z., Han, P., and Gu, J.-D. (2014). New PCR primers based on *mcrA* gene for retrieving more anaerobic methanotrophic archaea from coastal reedbed sediments. Appl. Microbiol. Biot*.* 98**,** 4663-4670. doi: 10.1007/s00253-014-5599-5.
